# miR-29b restrains cholangiocarcinoma progression by relieving DNMT3B-mediated repression of CDKN2B expression

**DOI:** 10.18632/aging.202549

**Published:** 2021-02-17

**Authors:** Kun Cao, Bo Li, Ye-Wei Zhang, Hui Song, Yi-Gang Chen, Yong-Jun Gong, Hai-Yang Li, Shi Zuo

**Affiliations:** 1 Department of Hepatobiliary Surgery, The Hospital Affiliated to Guizhou Medical University, Guiyang, Guizhou, P. R. of China; 2 Key Laboratory of Endemic and Ethnic Diseases of the Ministry of Education of P. R. China, Guizhou Medical University, Guiyang, Guizhou, P. R. of China

**Keywords:** cholangiocarcinoma, miR-29b, DNMT3B, methylation, CDKN2B

## Abstract

Numerous studies have reported the important role of microRNAs (miRNAs) in human cancers. Although abnormal miR-29b expression has been linked to tumorigenesis in several cancers, its role in cholangiocarcinoma remains largely unknown. We found that miR-29b expression is frequently downregulated in human cholangiocarcinoma QBC939 cells and in clinical tumor samples. In cholangiocarcinoma patients, low miR-29b expression predicts poor overall survival. Overexpression of miR-29b in QBC939 cells inhibited proliferation, induced G1 phase cycle arrest, and promoted apoptosis. Methylation-specific PCR (MSP) analysis revealed a decreased methylation imprint at the promoter of the cell cycle inhibitor gene CDKN2B in cells overexpressing miR-29b. After identifying the DNA methyltransferase DNMT3B as a putative miR-29b target, luciferase reporter assays confirmed a suppressive effect of miR-29b on DNMT3B expression. Accordingly, we detected an inverse correlation between miR-29b and DNMT3B expression in clinical cholangiocarcinoma specimens. In QBC939 cells, DNMT3B overexpression promoted proliferation and inhibited apoptosis. DNMT3B silencing, in turn, led to increased CDKN2B expression. We also observed significant growth arrest in subcutaneous tumors formed in nude mice by QBC939 cells overexpressing miR-29b. These findings suggest miR-29b functions as a tumor suppressor in cholangiocarcinoma by relieving DNMT3B-mediated repression of CDKN2B expression.

## INTRODUCTION

Cholangiocarcinoma is an extremely malignant tumor arising from cholangiocytes and epithelial cells lining the intra- and extra-hepatic biliary ducts [1]. Due to lack of sensitive indicators, cholangiocarcinoma patients are often diagnosed with late-stage disease and show a median overall survival of less than 12 months [2]. Despite advancements in surgical techniques and radiochemotherapy, the 5-year survival rate of cholangiocarcinoma patients is still approximately 20-40% [3]. Therefore, it is urgently necessary to decipher the molecular mechanisms underlying cholangio carcinoma progression to discover new biomarkers for diagnosis and treatment.

MicroRNAs (miRNAs) are short (about 22 nucleotides in length) non-coding RNAs that bind complementary sequences on target RNA transcripts and repress their expression by decreasing their stability or limiting their translation into protein [4]. Recent studies have demonstrated that miRNAs play pivotal roles in a variety of human diseases, including cancer [5]. Abnormally expressed miRNAs have been broadly implicated in cholangiocarcinoma pathogenesis [6–10]. miR-29b has been reported to be decreased in various cancers, where it functions as a tumor suppressor by inhibiting tumor cell proliferation and invasion, angiogenesis, and chemoresistance [11–13]. However, the involvement of miR-29b in cholangiocarcinoma development in not fully understood.

In the present study, we investigated miR-29b expression in a human cholangiocarcinoma cell line and in clinical tumor samples, and conducted *in vitro* and *in vivo* experiments to assess its role in cholangiocarcinoma progression. Our findings revealed that miR-29b has tumor suppressor activity linked to enhanced expression of the cell cycle inhibitor CDKN2B via suppression of the DNA methyltransferase DNMT3B. These data suggest that miR-29b may represent a novel prognostic biomarker and a potential therapeutic target for cholangiocarcinoma treatment.

## RESULTS

### miR-29b downregulation is associated with poor overall survival in cholangiocarcinoma patients

**Figure 1:**
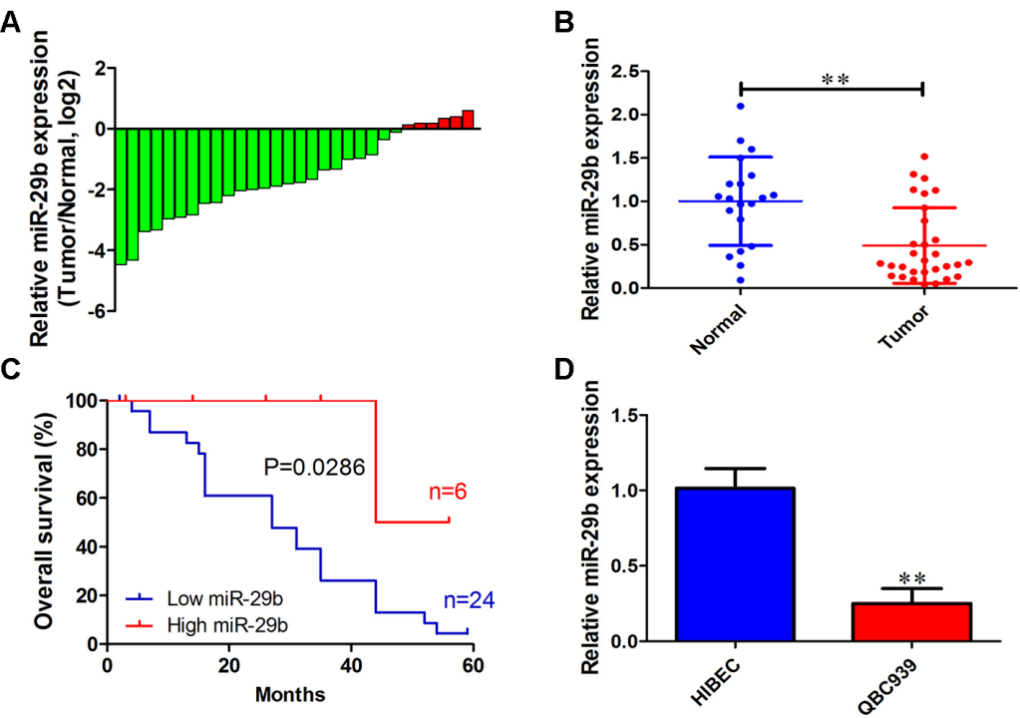
**miR-29b downregulation is associated with poor overall survival in cholangiocarcinoma.** (**A**) Relative miR-29b expression in 30 patients with cholangiocarcinoma after normalization to adjacent, normal tissue expression (**B**) miR-29b expression data for 30 cholangiocarcinoma and 20 non-tumor specimens. (**C**) Kaplan-Meier curves for cholangiocarcinoma patients with low and high expression of miR-29b. (**D**) Relative expression of miR-29b in the human cholangiocarcinoma cell line QBC939 and in human intrahepatic biliary epithelial cells (HIBEC). The values presented are means ± SD. *P<0.05 and **P<0.01 compared to the normal tissues or HIBEC cells, as determined by analysis of Student’s t-test.

To investigate the functional role of miR-29b in cholangiocarcinoma progression, we first determined miR-29b expression in 30 cholangiocarcinoma tissues and 20 adjacent normal tissues. qRT-PCR results showed that miR-29b expression was downregulated in most cholangiocarcinoma samples (24/30, 80%) exhibiting a mean 2.14-fold decrease relative to normal tissues (Figure 1A and 1B). In turn, Kaplan-Meier analysis revealed that low miR-29b expression was strongly correlated with unfavorable overall survival of cholangiocarcinoma patients (P=0.0286; Figure 1C). Consistently, miR-29b expression was also decreased in the human cholangiocarcinoma cell line QBC939, in comparison to human intrahepatic biliary epithelial cells (HIBEC) (Figure 1D). These results indicate that miR-29b might function as a tumor suppressor in cholangiocarcinoma.

### Overexpression of miR-29b suppresses proliferation and induces cell cycle arrest and apoptosis in cholangiocarcinoma cells

**Figure 2:**
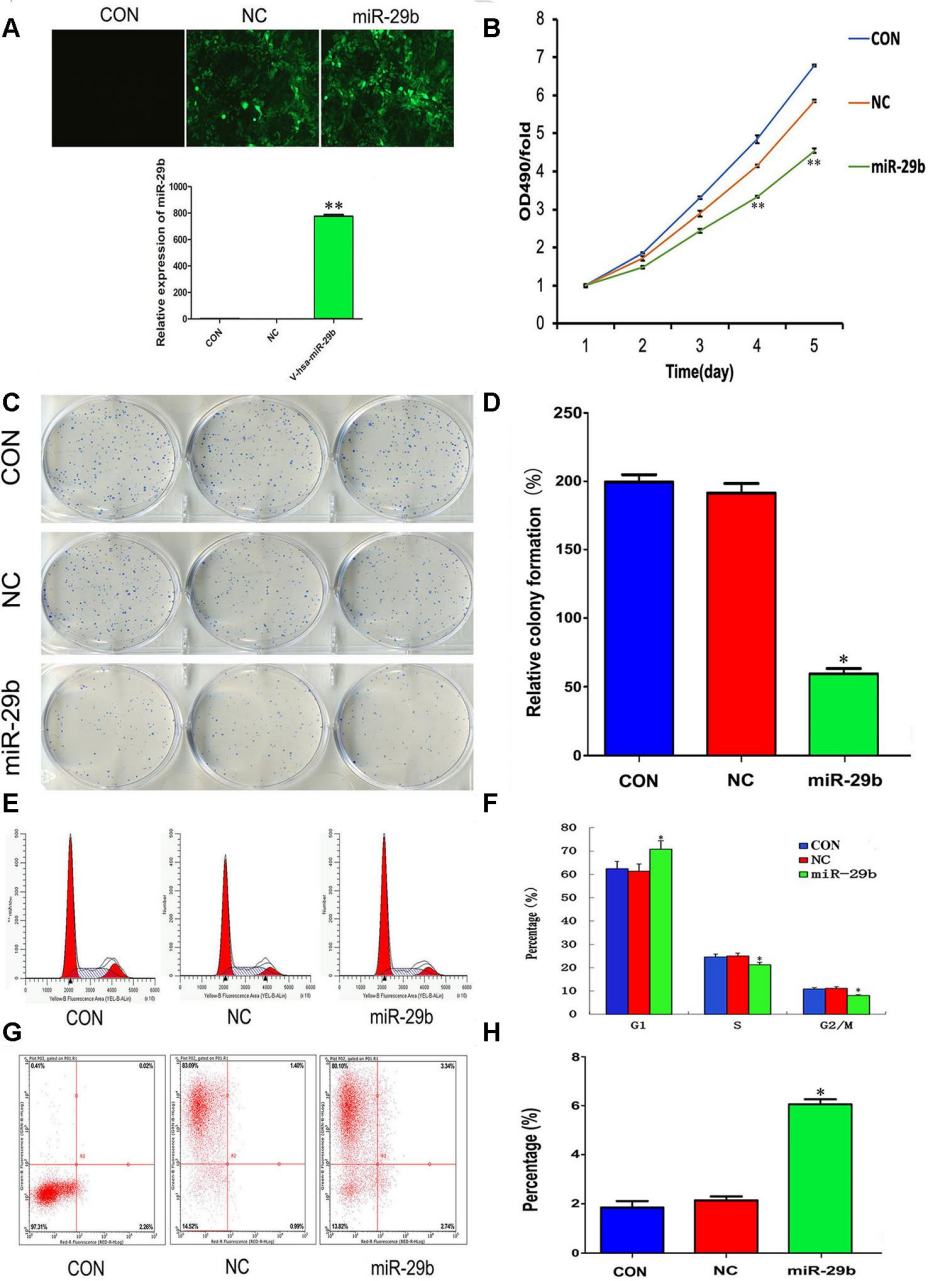
**Overexpression of miR-29b suppresses proliferation and induces cell cycle arrest and apoptosis in cholangiocarcinoma cells.** (**A**) Top: Representative fluorescence images of untransfected (CON), LV-miR-NC-transfected (NC), and LV-miR-29b-transfected (miR-29b) QBC939 cells (original magnification: 100×). Bottom: Relative miR-29b expression detected by qRT-PCR. (**B**) MTT assay results showing the time-course effect of miR-29b overexpression on the proliferation of QBC939 cells. (**C**) Representative images from colony formation assays. (**D**) Relative colony formation percentage. (**E**) Cell cycle distribution was subjected by flow cytometry. (**F**) Quantified histograms display the effect of miR-29b overexpression on cell cycle distribution. (**G**) Flow cytometry plots illustrating apoptosis in Annexin V/PI-stained QBC939 cells. (**H**) Quantified histograms display the effect of miR-29b overexpression on the apoptosis of QBC939 cells. The values presented are means ± SD. *P<0.05 and **P<0.01compared to the negative control group, as determined by analysis one-way variance (ANOVA), followed by the repeated measures.

To evaluate the effect of miR-29b on the proliferation of cholangiocarcinoma cells, a lentiviral vector overexpressing miR-29b (LV-miR-29b) or its corresponding negative control (LV-miR-NC) were alternatively transfected into QBC939 cells. After qRT-PCR validation (Figure 2A), we conducted MTT assays which showed decreased proliferation in miR-29b-overexpressing cells (Figure 2B). Moreover, a similar effect was observed in colony forming assays (Figure 2C and 2D). We also assessed the influence of miR-29b on both cell cycle distribution and apoptosis using flow cytometry. The results demonstrated that miR-29b overexpression led to significant accumulation of cells in G1 phase (Figure 2E and 2F) and a marked increase in the apoptotic rate (Figure 2G and 2H), compared with LV-miR-NC-transfected cells. These data suggest that miR-29b inhibits cholangiocarcinoma cell proliferation through induction of G1 phase cycle arrest and apoptosis.

### miR-29b prevents CDKN2B promoter methylation by targeting DNMT3B

**Figure 3:**
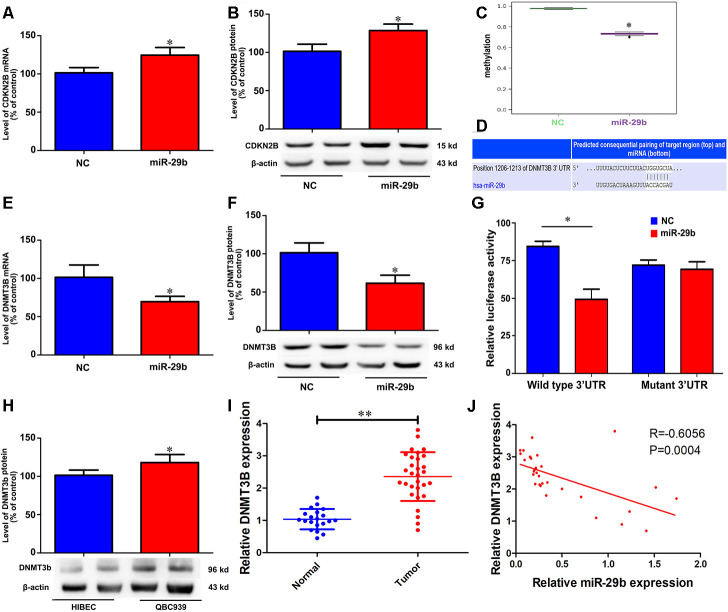
**miR-29b reduces CDKN2B promoter methylation and increases CDKN2B expression by directly targeting DNMT3B.** Effect of miR-29b overexpression on CDKN2B mRNA (**A**) and protein (**B**) levels in QBC939 cells. (**C**) MSP analysis of CDKN2B gene promoter methylation changes in QBC939 cells transfected with LV-miR-29b or LV-miR-NC. (**D**) Schematic representation of the putative miR-29b binding site in the DNMT3B 3’UTR. (**E**, **F**) Effect of miR-29b overexpression on DNMT3B mRNA (**E**) and protein (**F**) levels. (**G**) Dual-luciferase reporter assay results showing reduced WT-DNMT3B promoter-driven luciferase activity in QBC939 cells transfected with miR-29b mimics. (**H**) Relative expression of DNMT3B protein in QBC939 cells. (**I**) Relative expression of DNMT3B mRNA in clinical cholangiocarcinoma specimens. (**J**) Pearson correlation analysis between miR-29b and DNMT3B expression in cholangiocarcinoma specimens. The values presented are means ± SD. *P<0.05 compared to the negative control group, as determined by analysis of one-way variance (ANOVA), followed by the repeated measures.

Cyclin-dependent kinase inhibitor 2B (CDKN2B, also known as p15), is a well-known tumor suppressor that is deregulated in various tumors [14–16]. To test whether CDKN2B expression mediates the inhibitory effect of miR-29b overexpression on the proliferation of QBC939 cells, qRT-PCR and western blots were performed to determine CDKN2B levels. The results showed that miR-29b overexpression significantly increased CDKN2B expression at both the mRNA and protein levels (Figure 3A and 3B). To assess whether this effect involved changes in the methylation status of the CDKN2B gene promoter, we conducted methylation-specific PCR (MSP) assays. Consistent with the observed increase in CDKN2B expression, MSP results revealed that CDKN2B promoter methylation levels were reduced after transfection with LV-miR-29b (Figure 3C). These findings suggest that miR-29b inhibits the proliferation of cholangiocarcinoma cells by upregulating CDKN2B expression through demethylation of its promoter.

To uncover the mechanism through which miR-29b reduces the methylation imprint of the CDKN2B gene promoter, we performed bioinformatics analysis to identify DNA methyltransferases targeted by miR-29b. Results showed that the 3’UTR of DNA methyltransferase 3B (DNMT3B) contained a putative binding site for miR-29b (Figure 3D). Subsequently, we verified through qRT-PCR and western blot assays that LV-miR-29b transfection decreased both mRNA and protein levels of DNMT3B in QBC939 cells (Figure 3E and 3F). Accordingly, luciferase reporter assays showed that transfection of miR-29b mimics significantly decreased luciferase activity driven by a WT-DNMT3B vector, while a Mut-DNMT3B vector had no significant effect (Figure 3G). Meanwhile, western blot assays showed that DNMT3B levels were significantly higher in both QBC939 cells and clinical cholangiocarcinoma samples, compared to HIBEC and normal tissues, respectively (Figure 3H and 3I). Accordingly, a significant, inverse correlation between miR-29b and DNMT3B expression was detected through qRT-PCR analyses of clinical cholangiocarcinoma specimens (Figure 3J). These results indicate that DNMT3B is a direct target of miR-29b in cholangiocarcinoma.

### DNMT3B overexpression promotes cell proliferation and inhibits cell cycle arrest and apoptosis in cholangiocarcinoma cells

**Figure 4:**
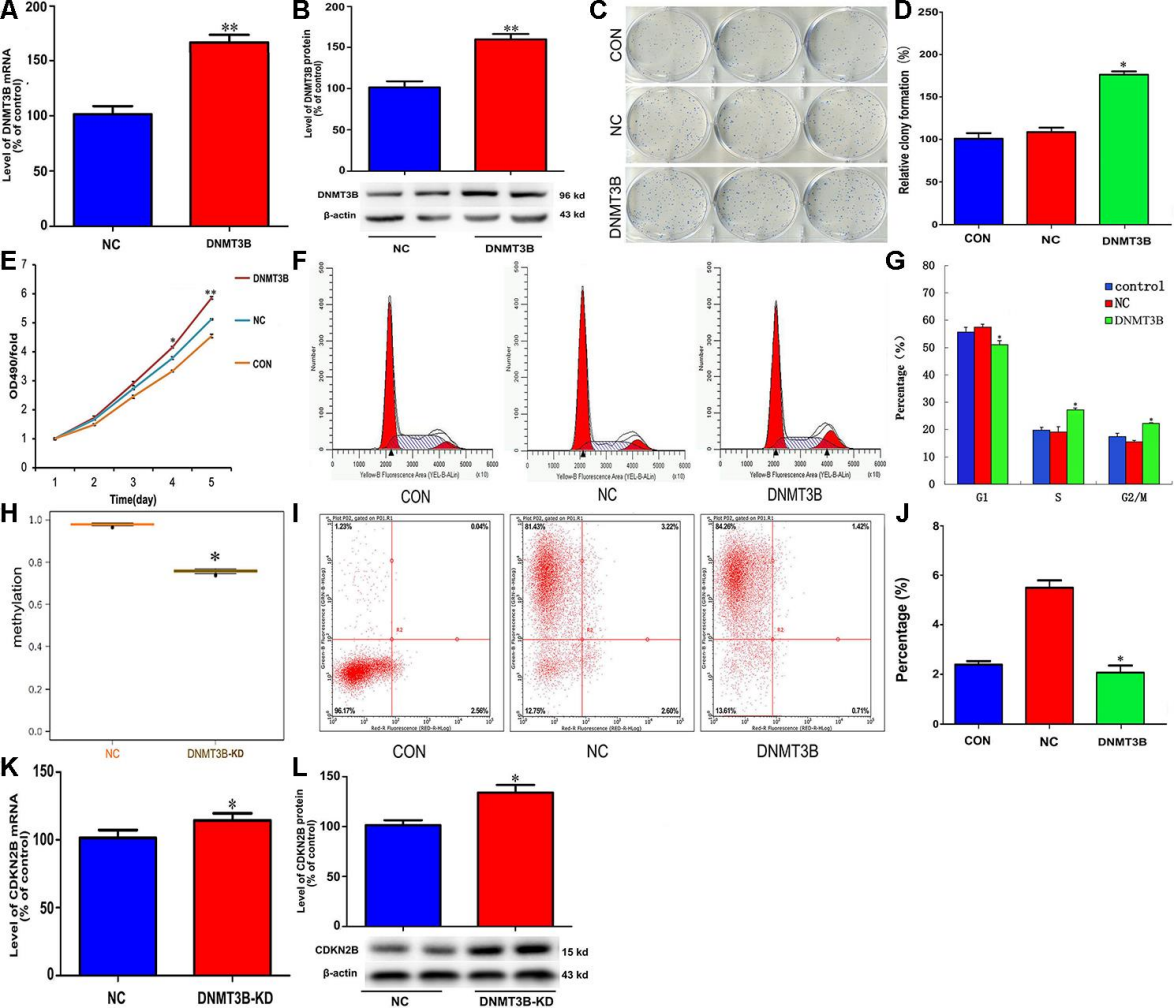
**DNMT3B overexpression promotes proliferation and inhibits cell cycle arrest and apoptosis in cholangiocarcinoma cells.** Analysis of DNMT3B mRNA (**A**) and protein (**B**) levels in QBC939 cells transfected with LV-DNMT3B or LV-control. (**C**–**E**) Effect of DNMT3B overexpression on QBC939 cell colony formation (**C** and **D**) and proliferation (**E**). (**F**) Cell cycle distribution was subjected by flow cytometry. (**G**) Quantified histograms display the effect of DNMT3B overexpression on cell cycle distribution. (**H**) Relative CDKN2B gene promoter methylation level in QBC939 cells transfected with LV-DNMT3B or LV-control. (**I**) Flow cytometry plots illustrating apoptosis in Annexin V/PI-stained QBC939 cells. (**J**) Quantified histograms display the effect of DNMT3B overexpression on the apoptosis of QBC939 cells. (**K**–**L**) Effect of DNMT3B knockdown on CDKN2B mRNA (**K**) and protein (**L**) levels. The values presented are means ± SD. *P<0.05 compared to the negative control group, as determined by analysis of one-way variance (ANOVA), followed by the repeated measures.

To explore the potential function of DNMT3B in cholangiocarcinoma pathogenesis, we transfected QBC939 cells with a lentiviral vector carrying the DNMT3B gene (LV-DNMT3B) or a negative control (LV-control) (Figure 4A and 4B). DNMT3B overexpression significantly increased colony formation and cell proliferation (Figure 4C–4E). Meanwhile, flow cytometry analyses revealed that compared to the effect of LV-control transfection, DNMT3B overexpression inhibited G1 phase cycle arrest and reduced apoptosis (Figure 4F–4J). Through MSP analysis, a decrease in CDKN2B promoter methylation was detected after short hairpin RNA-mediated DNMT3B knockdown (Figure 4H). Accordingly, DNMT3B silencing led to increased expression of CDKN2B mRNA and protein (Figure 4K and 4L). These data suggest that DNMT3B levels regulate cell cycle progression by effecting changes in the methylation status of the CDKN2B gene promoter to modulate its expression.

### Overexpression of miR-29b inhibits cholangiocarcinoma xenograft growth

**Figure 5:**
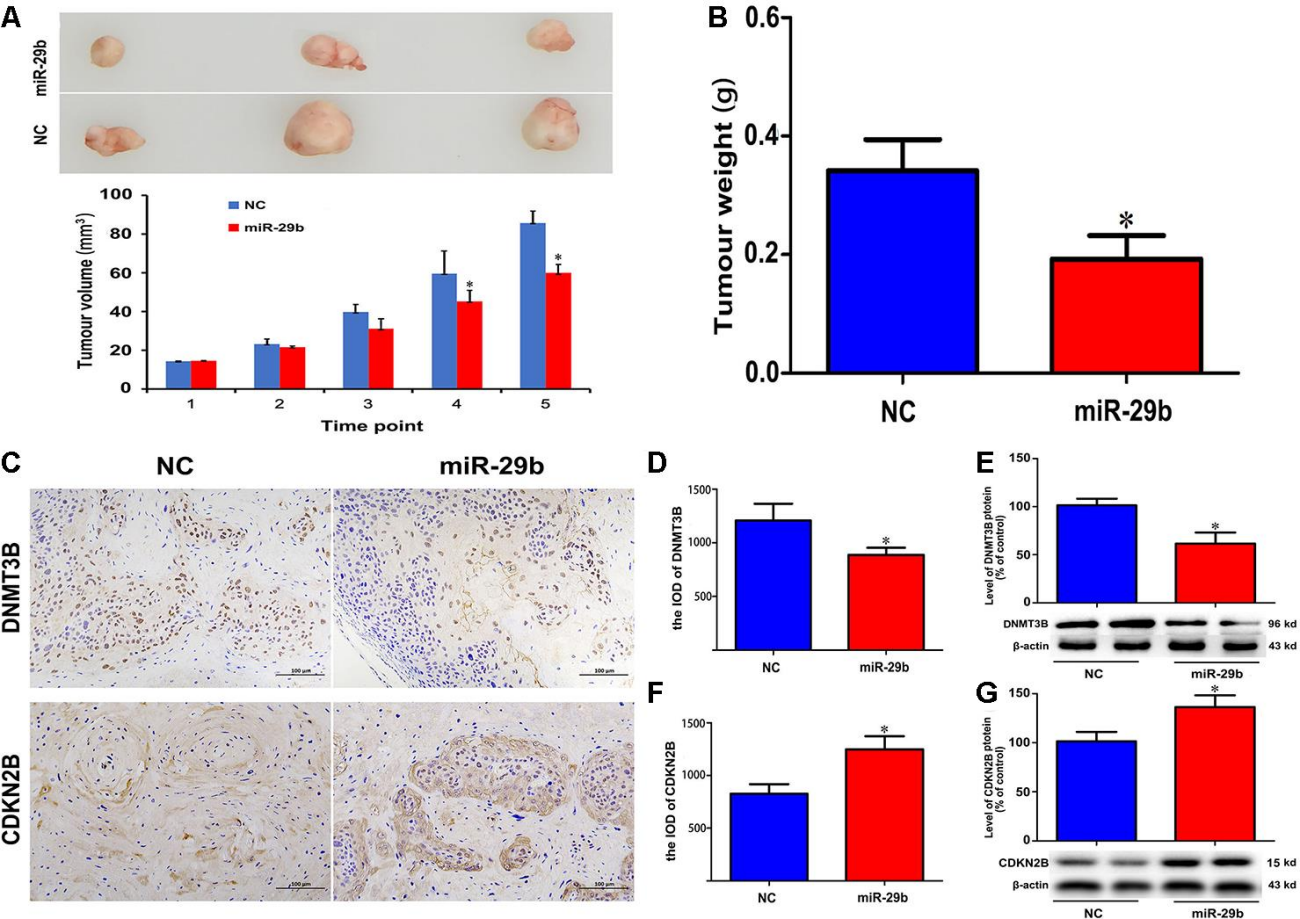
**miR-29b overexpression inhibits cholangiocarcinoma tumorigenesis *in vivo*.** (**A**) Representative images of subcutaneous tumors excised from nude mice (day 63 post-inoculation) and tumor volume curve. Measurements began on day 15 post-implantation and were repeated every 7 days until sacrifice. (**B**) Tumor weight at sacrifice. (**C**) Representative images of immunohistochemical analysis of DNMT3B and CDKN2B expression in excised tumors. (**D**–**G**) Corresponding semiquantitative expression analyses of DNMT3B and CDKN2B. DNMT3B mRNA (**D**) and protein (**E**) levels. CDKN2B mRNA (**F**) and protein (**G**) levels. Scale bars = 100 μm. The values presented are means ± SD. *P<0.05 compared to the negative control group, as determined by analysis of one-way variance (ANOVA), followed by the repeated measures.

To further confirm the inhibitory effect of miR-29b on cholangiocarcinoma tumorigenesis, QBC939 cells stably overexpressing miR-29b were subcutaneously inoculated into nude mice. As shown in Figure 5A, 5B, the resulting tumors demonstrated significant growth delay compared to tumors formed by control cells. Following tumor excision, immunohistochemical analysis showed decreased DNMT3B and increased CDKN2B expression in tumors overexpressing miR-29b (Figure 5C–5G). Taken together, these results suggest that miR-29b acts as a tumor suppressor in cholangiocarcinoma by downregulating DNMT3B and promoting the expression of CDKN2B.

## DISCUSSION

Accumulating evidence has revealed that miRNAs play important roles as either oncogenes or tumor suppressors during carcinogenesis [17–21]. Thus, unraveling the expression profile and function of individual miRNAs should contribute to the identification of useful diagnostic/prognostic markers and therapeutic targets for cancer treatment. In this study, we found that miR-29b was downregulated both in human cholangiocarcinoma QBC939 cells and clinical specimens, and this expression pattern was significantly associated with poor overall survival. These findings suggest that miR-29b acts as a tumor suppressor in cholangiocarcinoma.

Applying gain-of-function approaches, we demonstrated that overexpression of miR-29b induced G1 arrest, inhibited proliferation, and promoted apoptosis in cultured cholangiocarcinoma cells. Previous studies have showed that miR-29b interacts with multiple targets to exert tumor suppressor activity. For example, miR-29b overexpression suppressed proliferation, migration, and invasion of non-small cell lung cancer cells by reducing Striatin 4 (STRN4) expression [22]. In colon cancer, miR-29b inhibited cell growth and chemoresistance to oxaliplatin via targeting FOLR1 [23]. Wang et al. reported that miR-29b restrained gastric cancer cell migration and tumor growth through suppression of MMP2 expression [24]. In triple-negative breast cancer cells, however, miR-29b seems to have a more complex role [25–26].

DNA methylation is an important regulatory mechanism of gene transcription in mammalian cells [27]. Accordingly, aberrant or accidental methylation of the promoter of tumor-related genes has been observed in a wide variety of malignancies [28]. Promoter hypomethylation usually leads to gene activation [29]. In the present study, we identified DNMT3B as a downstream target of miR-29b, a fact reflected by the inverse expression pattern detected in clinical cholangiocarcinoma samples for these two genes. Accordingly, overexpression of DNMT3B significantly promoted cell proliferation and inhibited apoptosis in QBC939 cells. DNMT3B, a de novo DNA methyltransferase, is frequently increased in many malignancies [30]. This enzyme was reported to be essential for gene remethylation and might be a promising therapeutic target to prevent cancer progression [31–33]. We observed that miR-29b overexpression reduced methylation at the promoter of the CDKN2B gene, a cell growth regulator that inhibits cell cycle G1 progression, and this effect was mimicked by DNMT3B knockdown. Therefore, our data suggest that DNMT3B acts as an oncogene in cholangiocarcinoma.

In conclusion, our data revealed that miR-29b acts as a tumor suppressor in cholangiocarcinoma by promoting CDKN2B demethylation and transcription via suppression of DNMT3B activity. These findings highlight miR-29b as a promising diagnostic marker and potential therapeutic target for the treatment of cholangiocarcinoma and other cancers.

## MATERIALS AND METHODS

### Human tissue samples and cell culture

This study was approved by the research ethics committee of the Affiliated Hospital of Guizhou Medical University (NO. 2018005). Written informed consent was obtained from all patients. A total of 30 cholangiocarcinoma tissues and 20 adjacent tissues was obtained from cholangiocarcinoma patients undergoing surgical treatment. The cholangiocarcinoma cell line QBC939 was kindly provided by Professor Shuguang Wang of Third Military Medical University. Human intrahepatic biliary epithelial cells (HIBEC) were purchased from the Cell Bank of Shanghai Institutes for Biological Sciences, Chinese Academy of Sciences (Shanghai, China). Cells were cultured in DMEM medium with 10 % FBS and maintained at 37° C in a humidified incubator with 5 % CO_2_.

### RNA oligonucleotides and cell transfection

miR-29b mimics, miRNA negative control, short hairpin RNA specific for DNMT3B (sh-DNMT3B), and negative control shRNA (sh-NC) were synthesized by GenePharma (Shanghai, China). For gene overexpression experiments, the human miR-29b precursor and the full-length DNMT3B coding region were amplified using PCR and cloned into GV309 lentiviral vectors (LV-miR-29b and LV-DNMT3B). These constructs, as well as their negative controls, were generated by Genechem (Shanghai, China). Transfections of RNA oligonucleotides were performed using Lipofectamine 2000 reagent (Invitrogen, USA).

### Cell proliferation assay

Cells (2 × 10^3^ cells/well) were seeded into 96-well plates. At the indicated time points, 20 μl of MTT (5 g /L) was added into each well and incubated in the dark for 4 h. Then 150 μL of dimethylsulfoxide was added to each well. The absorbance of each well was measured at 490 nm (A490) on a spectrophotometer.

### Colony formation assay

Cells (500 cells/well) were seeded into 6-well plates and cultured for 2 weeks until visible colonies formed. The colonies were fixed in paraformaldehyde, stained with crystal violet solution, and total colony numbers per well were counted.

### Cell cycle analysis

After 48-h transfection, the cells were harvested and fixed in 75% ethanol at -20° C for 24 h. After being washed with PBS, the cells were stained by incubation in PBS containing 10 μg/mL propidium iodide (PI) and 0.5 mg/mL RNase A for 15 min at 37° C. Cell cycle analysis was performed using a FACSCalibur instrument (Becton Dickinson, USA).

### Apoptosis assay

Cell apoptosis was detected using an Annexin V-FITC apoptosis detection kit (Invitrogen, USA) according to the manufacturer’s protocols. After 48-h transfection, cells were harvested and dual-stained with 5 μl Annexin V and 5 μl PI for 30 min at room temperature. The stained cells were immediately analyzed by flow cytometry.

### qRT-PCR

Total RNA was extracted from tissues and cells with TRIzol (Invitrogen) and reversely transcribed to cDNA using the All-in-One First-Strand cDNA Synthesis kit (GeneCopoeia Inc., USA). The following primers were used: miR-29b Forward: 5'-GGAAAGGACGAAACACCGGCTAGGTTGTCTTGGGTTTATTG- 3', Reverse: 5'- TGTCTCGAGGTCGAGAATTAAAAAACTTCAGAGCTGTCCCATTCAC-3'. DNMT3B Forward: 5’- AGGGAAGACTCGATCCTCGTC-3’ Reverse: 5’-GTGTGTAGCTTAGCAGACTGG-3’. β-actin Forward: 5’-GGCATCCTCACCCTGAAGTA-3’Reverse: 5’-TAGCACAGCCTGGATAGCAA-3’. qRT-PCR analysis was performed on an ABI 7500HT System using SYBR Premix Ex Taq II (TaKaRa, Beijing, China). U6 or β-actin were used as normalization controls and relative expression levels calculated using the 2^-∆∆CT^ method.

### MSP assay

Genomic DNA was extracted from cells by standard phenol/chloroform extraction. DNA was quantified using a NanoDrop 2000 device (Thermo Fisher Scientific, USA). Genomic DNA (500 ng per sample) was bisulfite-converted using an EZ DNA Methylation-GOLD Kit according to manufacturer’s protocol (Zymo Research, USA). Briefly, DNA was bisulfite-converted for 2 h at 64° C and subsequently desulfonated, washed, and eluted in 10 μl elution buffer. Touchdown PCR was used to amplify the bisulfite-treated DNA. PCR primers were designed to amplify the CDKN2B promoter region in bisulfite-converted gDNA.

PCR primer design was performed using MethPrimer. After PCR amplification, indexed libraries of purified PCR products were generated using TruSeq DNA PCR-free library preparation technology according to manufacturer’s protocol (Illumina, USA). Denatured and diluted libraries were sequenced on an Illumina MiSeq benchtop sequencer with the sequencing-by-synthesis technology per manufacturer’s protocol (Illumina). The Illumina sequencing results were mapped to the genome using Bismark software. MethylKITR package software was then used to obtain site-specific methylation information. Finally, the methylation of each CpG site was defined as the number of methylated reads divided by the number of methylated and unmethylated reads combined. Results are expressed as the mean methylation over all CpGs and reads per sample and per amplicon.

### *In vivo* tumor growth assay

QBC939 cells stably overexpressing LV-miR-29b or control LV-miR-NC vector were established by selection with puromycin (Sigma, USA). A total of 5×10^6^ cells were then inoculated subcutaneously into 4-week-old female BALB/c mice (n = 6 per group). Starting on day 15, tumor size was measured every 7 days. After 63 days, mice were sacrificed, and tumors were dissected and weighed. All animal experimental procedures were performed in accordance with the guidelines of the Animal Ethical and Experimental Committee of the Affiliated Hospital of Guizhou Medical University.

### Immunohistochemistry analysis

Paraffin-embedded tissues obtained from mouse xenografts were sectioned to a thickness of 4 μm. Subsequently, sections were probed with anti-CDKN2B (GTX, #55855, 1:100 dilution) or anti-DNMT3B antibodies (GTX#129127; 1:100 dilution) at 4° C for overnight, incubated with suitable HRP-conjugated antibodies, and stained with diaminobenzidine tetrahydrochloride and hematoxylin as previously described [34]. Estimation of IOD (Integrated Optical Density) for each immunohistochemical staining was performed in 3 randomly selected microscopic fields per sample (original magnification 100× or 200×) using Image Pro Plus software (Media Cybernetics, USA).

### Western blotting

Cells were harvested and lysed with RIPA buffer. Aliquots containing 30 μg of protein were separated by SDS-PAGE and transferred onto PVDF membranes (Millipore, Bedford, MA). Membranes were blocked with 5% non-fat dry milk for 1 h at room temperature and then incubated with the following specific antibodies: CDKN2B (CST, #36303), DNMT3B (CST, #57868) or β-actin (CST, #3700) overnight at 4° C, followed by incubation with a HRP-conjugated secondary antibody at room temperature for 1 h. Bands were detected by chemiluminescence (Millipore, USA).

### Luciferase reporter assay

Wild type (WT) and mutant (Mut) DNMT3B 3’UTRs containing the predicted miR-29b target sites were synthesized by GenePharma and then cloned into pmirGLO vectors (Promega, USA). Cells (1 × 10^4^ cells/well) were seeded into 24-well plates. After 24-h incubation, miR-29b mimics or negative control miRNAs were co-transfected with WT-DNMT3B or Mut-DNMT3B vectors into cells. Luciferase activity was measured 48 h after transfection using a Dual-Luciferase Reporter Assay kit (Promega).

### Statistical analysis

All experiments were independently performed at least three times. Data are shown as the mean ± SD. Differences between groups were evaluated using Student’s t-test and one-way ANOVA with repeated measures. Kaplan-Meier analysis were used to analyze the impact of miR-29b on cholangiocarcinoma patients. The correlation between the expression of miR-29b and DNMT3B was performed employing the Pearson correlation test. Statistical analyses were conducted using GraphPad Prism 6.0. *P* <0.05 was considered significant.

### Ethics approval

Protocols for human tissue provision and animal experiments were approved by the Ethical Committee of the Hospital Affiliated to Guizhou Medical University, China (No. 2018005).
